# 3,3,9,9-Tetra­phenyl-2,4,8,10-tetra­oxa­spiro­[5.5]undeca­ne

**DOI:** 10.1107/S1600536810043795

**Published:** 2010-10-31

**Authors:** Xiaoqiang Sun, Liang Chen, Yan Jiang, Zhengyi Li

**Affiliations:** a Key Laboratory of Fine Chemical Engineering, Changzhou University, Changzhou 213164, Jiangsu, People’s Republic of China

## Abstract

In the title compound, C_31_H_28_O_4_, the asymmetric unit contains two crystallographically independent mol­ecules. In these two mol­ecules, the four dihedral angles between each pair of phenyl rings on the same C atoms are 75.4 (1), 83.0 (1), 85.0 (1) and 80.4 (2)°. All of the nonplanar six-membered heterocycles adopt chair conformations. Inter­molecular C—H⋯π and weak C—H⋯O inter­actions link the mol­ecules and are effective in the stabilization of the crystal structure.

## Related literature

For general background to spiranes, see: Cismaş *et al.* (2005 ▶); Mihiş *et al.* (2008 ▶); Sun *et al.* (2010 ▶).
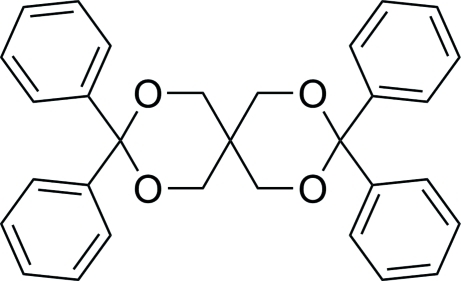


## Experimental

### 

#### Crystal data

C_31_H_28_O_4_
*M* *_r_* = 464.53
Triclinic, 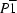

*a* = 13.509 (3) Å
*b* = 13.790 (3) Å
*c* = 15.626 (5) Å
α = 102.327 (7)°
β = 104.199 (6)°
γ = 112.170 (4)°
*V* = 2456.8 (11) Å^3^
*Z* = 4
Mo *K*α radiation
μ = 0.08 mm^−1^
*T* = 295 K
0.26 × 0.22 × 0.20 mm

#### Data collection

Bruker APEXII CCD diffractometer
Absorption correction: multi-scan (*SADABS*; Bruker, 2000 ▶) *T*_min_ = 0.979, *T*_max_ = 0.984
13730 measured reflections
8545 independent reflections
5355 reflections with *I* > 2σ(*I*)
*R*_int_ = 0.026

#### Refinement

*R*[*F* ^2^ > 2σ(*F* ^2^)] = 0.053
*wR*(*F* ^2^) = 0.196
*S* = 1.01
8545 reflections
631 parameters
1 restraint
H-atom parameters constrained
Δρ_max_ = 0.23 e Å^−3^
Δρ_min_ = −0.28 e Å^−3^

### 

Data collection: *SMART* (Bruker, 2000 ▶); cell refinement: *SAINT* (Bruker, 2000 ▶); data reduction: *SAINT*; program(s) used to solve structure: *SHELXTL* (Sheldrick, 2008 ▶); program(s) used to refine structure: *SHELXTL*; molecular graphics: *SHELXTL*; software used to prepare material for publication: *SHELXTL*.

## Supplementary Material

Crystal structure: contains datablocks I, global. DOI: 10.1107/S1600536810043795/bq2239sup1.cif

Structure factors: contains datablocks I. DOI: 10.1107/S1600536810043795/bq2239Isup2.hkl

Additional supplementary materials:  crystallographic information; 3D view; checkCIF report

## Figures and Tables

**Table 1:** Hydrogen-bond geometry (Å, °) *Cg*1 and *Cg*2 are the centroids of the C38–C43 and C7–C12 rings, respectively.

*D*—H⋯*A*	*D*—H	H⋯*A*	*D*⋯*A*	*D*—H⋯*A*
C9—H9⋯*Cg*1^i^	0.93	2.83	3.722 (2)	161
C30—H30⋯*Cg*2^ii^	0.93	2.84	3.523 (3)	131
C3—H3*A*⋯O6^ii^	0.97	2.53	3.436 (3)	156

Symmetry codes: (i) 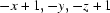; (ii) 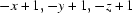.

**Fig. 1.**
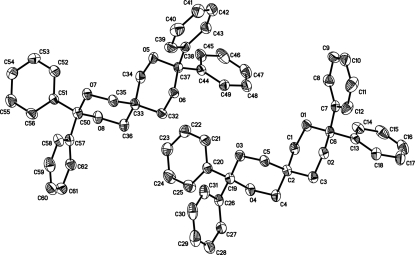
The molecular structure of the title compound (I). Displacement ellipsoids are drawn at the 30% probability level. All H atoms have been omitted for clarity.

## supplementary crystallographic information

### Comment

Owing to the characteristic axial and helical chirality, the stereochemistry of spiranes with six-membered rings has been extensively studied (Cismaş *et al.*, 2005). In the past three decades, most of these investigations were carried out with spiranes containing 1,3-dioxane units (Mihiş *et al.*, 2008; Sun *et al.*, 2010). We herein present the structure of 3,3,9,9-tetraphenyl-2,4,8,10-tetraoxaspiro[5.5]undecane (I).

In the crystal structure of (I), the asymmetric unit contains two crystallographically independent molecules (Fig. 1). In these two molecules, the four dihedral angles between each two phenyl rings on the same C atoms are all different. In one molecule, the dihedral angles formed by the phenyl rings (C7–C12) and (C13–C18) are 75.4 (1)° and 83.0 (1)° for the phenyl rings (C20–C25) and (C26–C31). In the other molecule, the dihedral angles between the phenyl rings (C38–C43) and (C44–C49) are 85.0 (1)° and 80.4 (2)° for the phenyl rings (C51–C56) and (C57–C62). The four nonplanar six-membered heterocycles [(O1/O2/C1–C3/C6), (O3/O4/C2/C4/C5/C19), (O5/O6/C32–C34/C37) and (O7/O8/C33/C35/C36/C50)] all adopt chair conformations.

Intermolecular C—H···π and weak C—H···O interactions link the molecules and are effective in the stabilization of the crystal structure (Table 1).

### Experimental

To a solution of benzophenone (7.3 mmol, 1.33 g) and pentaerythritol (4 mmol, 0.54 g) in toluene (30 ml), phosphotungstic acid (30 mg) as catalyst was added, respectively. The mixtures were refluxed for 4 h to complete the reaction. After reaction, the solvent was evaporated under vacuum and the resulting solid was washed with 5% sodium bicarboinate (20 ml) and 50% ethanol (20 ml). The product recrystallized from ethanol to afford a white solid (71% yield, m.p. 436–437 K). Single crystals suitable for X-ray diffraction were also obtained by evaporation of an ethanol solution.

### Refinement

All the H atoms were placed in geometrically idealized positions and constrained to ride on their parent atoms, with C—H distances of 0.93–0.97 Å, and with *U*_iso_(H) = 1.2*U*_eq_(C).

### Crystal data

**Table:**

C_31_H_28_O_4_	*Z* = 4
*M**_r_* = 464.53	*F*(000) = 984
Triclinic, *P*1	*D*_x_ = 1.256 Mg m^−^^3^
Hall symbol: -P 1	Melting point = 436–437 K
*a* = 13.509 (3) Å	Mo *K*α radiation, λ = 0.71073 Å
*b* = 13.790 (3) Å	Cell parameters from 13730 reflections
*c* = 15.626 (5) Å	θ = 2.6–24.8°
α = 102.327 (7)°	µ = 0.08 mm^−^^1^
β = 104.199 (6)°	*T* = 295 K
γ = 112.170 (4)°	Prism, colourless
*V* = 2456.8 (11) Å^3^	0.26 × 0.22 × 0.20 mm

### Data collection

**Table:**

Bruker APEXII CCD diffractometer	8545 independent reflections
Radiation source: fine-focus sealed tube	5355 reflections with *I* > 2σ(*I*)
graphite	*R*_int_ = 0.026
φ and ω scans	θ_max_ = 25.0°, θ_min_ = 1.4°
Absorption correction: multi-scan (*SADABS*; Bruker, 2000)	*h* = −14→16
*T*_min_ = 0.979, *T*_max_ = 0.984	*k* = −16→15
13730 measured reflections	*l* = −14→18

### Refinement

**Table:**

Refinement on *F*^2^	Primary atom site location: structure-invariant direct methods
Least-squares matrix: full	Secondary atom site location: difference Fourier map
*R*[*F*^2^ > 2σ(*F*^2^)] = 0.053	Hydrogen site location: inferred from neighbouring sites
*wR*(*F*^2^) = 0.196	H-atom parameters constrained
*S* = 1.01	*w* = 1/[σ^2^(*F*_o_^2^) + (0.102*P*)^2^ + 0.5251*P*] where *P* = (*F*_o_^2^ + 2*F*_c_^2^)/3
8545 reflections	(Δ/σ)_max_ < 0.001
631 parameters	Δρ_max_ = 0.23 e Å^−^^3^
1 restraint	Δρ_min_ = −0.28 e Å^−^^3^

### Special details

**Table:**

Geometry. All e.s.d.'s (except the e.s.d. in the dihedral angle between two l.s. planes) are estimated using the full covariance matrix. The cell e.s.d.'s are taken into account individually in the estimation of e.s.d.'s in distances, angles and torsion angles; correlations between e.s.d.'s in cell parameters are only used when they are defined by crystal symmetry. An approximate (isotropic) treatment of cell e.s.d.'s is used for estimating e.s.d.'s involving l.s. planes.
Refinement. Refinement of *F*^2^ against ALL reflections. The weighted *R*-factor *wR* and goodness of fit *S* are based on *F*^2^, conventional *R*-factors *R* are based on *F*, with *F* set to zero for negative *F*^2^. The threshold expression of *F*^2^ > σ(*F*^2^) is used only for calculating *R*-factors(gt) *etc*. and is not relevant to the choice of reflections for refinement. *R*-factors based on *F*^2^ are statistically about twice as large as those based on *F*, and *R*- factors based on ALL data will be even larger.

### Fractional atomic coordinates and isotropic or equivalent isotropic displacement parameters (Å^2^)

**Table:**

	*x*	*y*	*z*	*U*_iso_*/*U*_eq_	
C1	0.5250 (2)	0.5283 (2)	0.82950 (19)	0.0472 (7)	
H1A	0.5400	0.5658	0.8947	0.057*	
H1B	0.4457	0.4718	0.8013	0.057*	
C2	0.5446 (2)	0.6118 (2)	0.77849 (17)	0.0406 (6)	
C3	0.6715 (2)	0.6927 (2)	0.82180 (18)	0.0456 (7)	
H3A	0.6888	0.7465	0.7897	0.055*	
H3B	0.6910	0.7329	0.8874	0.055*	
C4	0.4723 (2)	0.6725 (2)	0.78827 (18)	0.0483 (7)	
H4A	0.4881	0.7069	0.8543	0.058*	
H4B	0.4925	0.7310	0.7611	0.058*	
C5	0.5090 (2)	0.5550 (2)	0.67320 (17)	0.0436 (6)	
H5A	0.5278	0.6110	0.6434	0.052*	
H5B	0.5509	0.5128	0.6626	0.052*	
C6	0.7171 (2)	0.5520 (2)	0.85862 (17)	0.0403 (6)	
C7	0.7748 (2)	0.4827 (2)	0.82801 (17)	0.0439 (6)	
C8	0.7288 (3)	0.3705 (2)	0.81755 (19)	0.0546 (7)	
H8	0.6618	0.3373	0.8291	0.066*	
C9	0.7821 (3)	0.3071 (3)	0.7899 (2)	0.0638 (9)	
H9	0.7514	0.2322	0.7840	0.077*	
C10	0.8781 (3)	0.3544 (3)	0.7717 (2)	0.0764 (11)	
H10	0.9124	0.3112	0.7519	0.092*	
C11	0.9263 (3)	0.4654 (3)	0.7817 (3)	0.0847 (12)	
H11	0.9932	0.4974	0.7697	0.102*	
C12	0.8742 (2)	0.5292 (3)	0.8100 (2)	0.0671 (9)	
H12	0.9066	0.6044	0.8170	0.080*	
C13	0.7661 (2)	0.6058 (2)	0.96562 (17)	0.0417 (6)	
C14	0.7190 (3)	0.5509 (3)	1.0212 (2)	0.0544 (7)	
H14	0.6522	0.4838	0.9932	0.065*	
C15	0.7706 (3)	0.5954 (3)	1.1181 (2)	0.0705 (10)	
H15	0.7382	0.5582	1.1549	0.085*	
C16	0.8688 (3)	0.6935 (4)	1.1597 (2)	0.0752 (11)	
H16	0.9028	0.7233	1.2248	0.090*	
C17	0.9178 (3)	0.7485 (3)	1.1057 (2)	0.0685 (9)	
H17	0.9855	0.8147	1.1342	0.082*	
C18	0.8662 (2)	0.7051 (2)	1.00877 (19)	0.0525 (7)	
H18	0.8990	0.7428	0.9724	0.063*	
C19	0.3205 (2)	0.5361 (2)	0.64462 (17)	0.0419 (6)	
C20	0.1985 (2)	0.4453 (2)	0.61579 (17)	0.0426 (6)	
C21	0.1780 (2)	0.3370 (2)	0.60770 (19)	0.0501 (7)	
H21	0.2386	0.3190	0.6185	0.060*	
C22	0.0671 (3)	0.2551 (3)	0.5835 (2)	0.0593 (8)	
H22	0.0539	0.1824	0.5779	0.071*	
C23	−0.0232 (3)	0.2806 (3)	0.5677 (2)	0.0604 (8)	
H23	−0.0974	0.2254	0.5514	0.072*	
C24	−0.0032 (3)	0.3877 (3)	0.5761 (3)	0.0725 (10)	
H24	−0.0642	0.4053	0.5658	0.087*	
C25	0.1072 (2)	0.4705 (3)	0.5999 (2)	0.0638 (8)	
H25	0.1199	0.5430	0.6051	0.077*	
C26	0.3259 (2)	0.6120 (2)	0.58554 (18)	0.0437 (6)	
C27	0.3225 (2)	0.7116 (2)	0.6153 (2)	0.0570 (8)	
H27	0.3221	0.7370	0.6753	0.068*	
C28	0.3198 (3)	0.7751 (3)	0.5571 (3)	0.0719 (10)	
H28	0.3168	0.8420	0.5779	0.086*	
C29	0.3214 (3)	0.7383 (4)	0.4689 (3)	0.0819 (12)	
H29	0.3208	0.7809	0.4301	0.098*	
C30	0.3240 (3)	0.6396 (4)	0.4381 (2)	0.0742 (10)	
H30	0.3238	0.6145	0.3778	0.089*	
C31	0.3270 (2)	0.5760 (3)	0.49551 (19)	0.0581 (8)	
H31	0.3296	0.5091	0.4739	0.070*	
C32	0.1745 (2)	0.2197 (2)	0.34555 (18)	0.0433 (6)	
H32A	0.1880	0.2826	0.3237	0.052*	
H32B	0.1858	0.2455	0.4116	0.052*	
C33	0.0521 (2)	0.1305 (2)	0.29122 (17)	0.0445 (6)	
C34	0.0392 (2)	0.0302 (2)	0.32237 (19)	0.0501 (7)	
H34A	0.0450	0.0488	0.3875	0.060*	
H34B	−0.0359	−0.0315	0.2848	0.060*	
C35	0.0254 (2)	0.0994 (2)	0.18545 (17)	0.0467 (7)	
H35A	0.0429	0.1660	0.1685	0.056*	
H35B	0.0731	0.0667	0.1686	0.056*	
C36	−0.0318 (2)	0.1720 (2)	0.31093 (18)	0.0487 (7)	
H36A	−0.0210	0.1887	0.3770	0.058*	
H36B	−0.0168	0.2401	0.2968	0.058*	
C37	0.2397 (2)	0.0832 (2)	0.36191 (17)	0.0420 (6)	
C38	0.3157 (2)	0.0397 (2)	0.32644 (17)	0.0429 (6)	
C39	0.3208 (3)	0.0369 (2)	0.2383 (2)	0.0599 (8)	
H39	0.2763	0.0604	0.2008	0.072*	
C40	0.3903 (3)	0.0001 (3)	0.2057 (2)	0.0724 (10)	
H40	0.3923	−0.0010	0.1464	0.087*	
C41	0.4571 (3)	−0.0350 (3)	0.2597 (2)	0.0697 (9)	
H41	0.5056	−0.0580	0.2380	0.084*	
C42	0.4512 (3)	−0.0357 (3)	0.3459 (2)	0.0676 (9)	
H42	0.4937	−0.0620	0.3820	0.081*	
C43	0.3824 (3)	0.0026 (2)	0.3794 (2)	0.0553 (7)	
H43	0.3809	0.0036	0.4387	0.066*	
C44	0.2730 (2)	0.1143 (2)	0.46846 (17)	0.0416 (6)	
C45	0.2173 (2)	0.0406 (3)	0.5081 (2)	0.0556 (8)	
H45	0.1542	−0.0270	0.4698	0.067*	
C46	0.2547 (3)	0.0667 (3)	0.6047 (2)	0.0718 (10)	
H46	0.2157	0.0176	0.6311	0.086*	
C47	0.3499 (3)	0.1655 (3)	0.6616 (2)	0.0682 (9)	
H47	0.3756	0.1827	0.7264	0.082*	
C48	0.4066 (3)	0.2383 (3)	0.6225 (2)	0.0601 (8)	
H48	0.4710	0.3048	0.6609	0.072*	
C49	0.3683 (2)	0.2132 (2)	0.52624 (18)	0.0481 (7)	
H49	0.4068	0.2632	0.5002	0.058*	
C50	−0.1712 (2)	0.0584 (2)	0.15671 (17)	0.0440 (6)	
C51	−0.2886 (2)	−0.0445 (2)	0.11423 (17)	0.0404 (6)	
C52	−0.2966 (3)	−0.1480 (2)	0.1128 (2)	0.0568 (8)	
H52	−0.2302	−0.1552	0.1350	0.068*	
C53	−0.4011 (3)	−0.2399 (3)	0.0791 (2)	0.0611 (8)	
H53	−0.4050	−0.3087	0.0791	0.073*	
C54	−0.4999 (3)	−0.2311 (3)	0.0454 (2)	0.0550 (7)	
H54	−0.5706	−0.2940	0.0215	0.066*	
C55	−0.4943 (3)	−0.1302 (3)	0.0469 (2)	0.0666 (9)	
H55	−0.5613	−0.1237	0.0254	0.080*	
C56	−0.3890 (2)	−0.0370 (3)	0.0805 (2)	0.0584 (8)	
H56	−0.3858	0.0315	0.0804	0.070*	
C57	−0.1705 (2)	0.1521 (2)	0.12028 (17)	0.0431 (6)	
C58	−0.1490 (2)	0.1561 (2)	0.03813 (19)	0.0498 (7)	
H58	−0.1306	0.1037	0.0072	0.060*	
C59	−0.1551 (3)	0.2387 (3)	0.0022 (2)	0.0657 (8)	
H59	−0.1411	0.2407	−0.0529	0.079*	
C60	−0.1812 (3)	0.3161 (3)	0.0467 (2)	0.0653 (8)	
H60	−0.1829	0.3721	0.0230	0.078*	
C61	−0.2053 (3)	0.3124 (3)	0.1271 (2)	0.0615 (8)	
H61	−0.2261	0.3639	0.1561	0.074*	
C62	−0.1984 (2)	0.2318 (2)	0.1642 (2)	0.0517 (7)	
H62	−0.2127	0.2308	0.2194	0.062*	
O1	0.59843 (14)	0.47657 (14)	0.82486 (12)	0.0465 (5)	
O2	0.73780 (14)	0.63359 (15)	0.81425 (12)	0.0444 (4)	
O3	0.38922 (14)	0.48245 (15)	0.63265 (12)	0.0457 (5)	
O4	0.35231 (15)	0.59668 (15)	0.74173 (11)	0.0466 (5)	
O5	0.12532 (15)	−0.00276 (14)	0.31258 (12)	0.0490 (5)	
O6	0.25474 (14)	0.17839 (14)	0.33426 (12)	0.0419 (4)	
O7	−0.09301 (15)	0.02172 (15)	0.13444 (12)	0.0459 (5)	
O8	−0.14763 (15)	0.09070 (15)	0.25580 (11)	0.0481 (5)	

### Atomic displacement parameters (Å^2^)

**Table:**

	*U*^11^	*U*^22^	*U*^33^	*U*^12^	*U*^13^	*U*^23^
C1	0.0328 (14)	0.0589 (17)	0.0487 (16)	0.0171 (13)	0.0154 (12)	0.0224 (13)
C2	0.0362 (14)	0.0513 (16)	0.0370 (14)	0.0206 (12)	0.0138 (11)	0.0181 (12)
C3	0.0413 (15)	0.0506 (16)	0.0435 (15)	0.0165 (13)	0.0140 (12)	0.0227 (12)
C4	0.0439 (16)	0.0586 (17)	0.0359 (14)	0.0226 (14)	0.0091 (12)	0.0113 (12)
C5	0.0361 (15)	0.0568 (17)	0.0447 (15)	0.0252 (13)	0.0170 (12)	0.0196 (12)
C6	0.0309 (14)	0.0481 (15)	0.0375 (14)	0.0125 (12)	0.0100 (11)	0.0190 (11)
C7	0.0372 (15)	0.0512 (17)	0.0349 (14)	0.0183 (13)	0.0063 (11)	0.0100 (12)
C8	0.0625 (19)	0.063 (2)	0.0440 (16)	0.0314 (16)	0.0175 (14)	0.0240 (14)
C9	0.083 (3)	0.064 (2)	0.0513 (18)	0.0458 (19)	0.0152 (17)	0.0192 (15)
C10	0.063 (2)	0.087 (3)	0.067 (2)	0.051 (2)	0.0045 (17)	−0.0031 (19)
C11	0.0421 (19)	0.084 (3)	0.098 (3)	0.0208 (19)	0.0225 (18)	−0.008 (2)
C12	0.0393 (17)	0.060 (2)	0.080 (2)	0.0125 (15)	0.0205 (15)	0.0016 (16)
C13	0.0407 (15)	0.0525 (16)	0.0403 (14)	0.0277 (13)	0.0149 (12)	0.0187 (12)
C14	0.0621 (19)	0.0673 (19)	0.0567 (18)	0.0411 (16)	0.0285 (15)	0.0335 (15)
C15	0.092 (3)	0.108 (3)	0.056 (2)	0.072 (3)	0.040 (2)	0.047 (2)
C16	0.085 (3)	0.120 (3)	0.0393 (17)	0.074 (3)	0.0155 (18)	0.0181 (19)
C17	0.055 (2)	0.085 (2)	0.0463 (18)	0.0338 (18)	0.0028 (15)	0.0015 (16)
C18	0.0445 (17)	0.0606 (18)	0.0441 (16)	0.0211 (15)	0.0115 (13)	0.0126 (13)
C19	0.0391 (15)	0.0537 (16)	0.0356 (13)	0.0237 (13)	0.0153 (11)	0.0131 (11)
C20	0.0413 (15)	0.0524 (17)	0.0374 (14)	0.0233 (13)	0.0151 (11)	0.0167 (12)
C21	0.0472 (17)	0.0583 (18)	0.0535 (17)	0.0306 (15)	0.0200 (13)	0.0208 (14)
C22	0.0523 (19)	0.0568 (19)	0.0614 (19)	0.0178 (16)	0.0198 (15)	0.0199 (15)
C23	0.0436 (18)	0.065 (2)	0.064 (2)	0.0179 (16)	0.0193 (14)	0.0200 (16)
C24	0.0417 (19)	0.086 (3)	0.098 (3)	0.0330 (18)	0.0246 (17)	0.039 (2)
C25	0.0396 (17)	0.067 (2)	0.094 (2)	0.0270 (16)	0.0272 (16)	0.0341 (18)
C26	0.0317 (14)	0.0541 (17)	0.0421 (15)	0.0168 (12)	0.0107 (11)	0.0188 (12)
C27	0.0503 (18)	0.0587 (19)	0.0546 (18)	0.0233 (15)	0.0085 (14)	0.0200 (15)
C28	0.0496 (19)	0.065 (2)	0.087 (3)	0.0193 (16)	0.0035 (17)	0.0360 (19)
C29	0.056 (2)	0.089 (3)	0.075 (3)	0.006 (2)	0.0052 (18)	0.052 (2)
C30	0.051 (2)	0.109 (3)	0.0482 (19)	0.018 (2)	0.0123 (15)	0.040 (2)
C31	0.0486 (18)	0.077 (2)	0.0415 (16)	0.0223 (16)	0.0139 (13)	0.0220 (14)
C32	0.0441 (16)	0.0445 (15)	0.0464 (15)	0.0224 (13)	0.0154 (12)	0.0214 (12)
C33	0.0438 (15)	0.0530 (16)	0.0360 (14)	0.0201 (13)	0.0121 (12)	0.0197 (12)
C34	0.0392 (16)	0.0552 (17)	0.0470 (16)	0.0134 (13)	0.0097 (12)	0.0225 (13)
C35	0.0450 (16)	0.0613 (18)	0.0394 (14)	0.0263 (14)	0.0167 (12)	0.0218 (13)
C36	0.0452 (16)	0.0615 (18)	0.0358 (14)	0.0223 (14)	0.0129 (12)	0.0153 (12)
C37	0.0365 (14)	0.0384 (14)	0.0429 (14)	0.0100 (12)	0.0078 (11)	0.0195 (11)
C38	0.0412 (15)	0.0386 (14)	0.0413 (15)	0.0130 (12)	0.0106 (12)	0.0135 (11)
C39	0.081 (2)	0.0571 (19)	0.0539 (18)	0.0398 (17)	0.0258 (16)	0.0251 (14)
C40	0.094 (3)	0.071 (2)	0.058 (2)	0.038 (2)	0.0380 (19)	0.0191 (17)
C41	0.061 (2)	0.079 (2)	0.068 (2)	0.0341 (19)	0.0256 (17)	0.0147 (18)
C42	0.065 (2)	0.075 (2)	0.059 (2)	0.0405 (18)	0.0102 (16)	0.0132 (16)
C43	0.0576 (19)	0.0617 (19)	0.0458 (16)	0.0298 (16)	0.0133 (14)	0.0175 (14)
C44	0.0391 (15)	0.0480 (16)	0.0392 (14)	0.0198 (13)	0.0126 (11)	0.0195 (12)
C45	0.0469 (17)	0.0644 (19)	0.0616 (19)	0.0238 (15)	0.0182 (14)	0.0372 (15)
C46	0.072 (2)	0.114 (3)	0.071 (2)	0.056 (2)	0.0407 (19)	0.063 (2)
C47	0.077 (2)	0.104 (3)	0.0431 (17)	0.060 (2)	0.0207 (17)	0.0271 (18)
C48	0.061 (2)	0.070 (2)	0.0457 (17)	0.0386 (17)	0.0067 (14)	0.0117 (15)
C49	0.0488 (17)	0.0480 (16)	0.0435 (15)	0.0204 (14)	0.0124 (12)	0.0158 (12)
C50	0.0434 (15)	0.0604 (18)	0.0342 (13)	0.0269 (14)	0.0176 (12)	0.0170 (12)
C51	0.0389 (15)	0.0508 (16)	0.0394 (14)	0.0242 (13)	0.0171 (11)	0.0194 (12)
C52	0.0471 (18)	0.060 (2)	0.0646 (19)	0.0289 (16)	0.0163 (14)	0.0202 (15)
C53	0.069 (2)	0.0461 (17)	0.065 (2)	0.0276 (17)	0.0197 (16)	0.0173 (14)
C54	0.0500 (18)	0.0530 (18)	0.0535 (17)	0.0174 (15)	0.0172 (14)	0.0151 (14)
C55	0.0443 (18)	0.068 (2)	0.080 (2)	0.0214 (16)	0.0124 (16)	0.0298 (17)
C56	0.0432 (17)	0.0532 (18)	0.081 (2)	0.0244 (15)	0.0169 (15)	0.0282 (16)
C57	0.0369 (15)	0.0509 (16)	0.0372 (14)	0.0164 (13)	0.0101 (11)	0.0176 (12)
C58	0.0445 (16)	0.0705 (19)	0.0444 (15)	0.0319 (15)	0.0171 (12)	0.0260 (14)
C59	0.061 (2)	0.086 (2)	0.0522 (18)	0.0287 (18)	0.0182 (15)	0.0382 (16)
C60	0.0502 (19)	0.065 (2)	0.080 (2)	0.0230 (16)	0.0130 (16)	0.0404 (17)
C61	0.0511 (19)	0.0553 (19)	0.073 (2)	0.0254 (15)	0.0137 (16)	0.0206 (16)
C62	0.0472 (17)	0.0599 (18)	0.0529 (17)	0.0292 (15)	0.0182 (13)	0.0187 (14)
O1	0.0319 (10)	0.0497 (11)	0.0537 (11)	0.0141 (9)	0.0122 (8)	0.0218 (9)
O2	0.0371 (10)	0.0552 (11)	0.0443 (10)	0.0183 (9)	0.0163 (8)	0.0264 (8)
O3	0.0372 (10)	0.0539 (11)	0.0467 (10)	0.0242 (9)	0.0140 (8)	0.0128 (8)
O4	0.0421 (11)	0.0633 (12)	0.0353 (9)	0.0256 (9)	0.0153 (8)	0.0137 (8)
O5	0.0378 (10)	0.0422 (10)	0.0499 (11)	0.0090 (9)	0.0044 (8)	0.0138 (8)
O6	0.0394 (10)	0.0444 (10)	0.0456 (10)	0.0186 (8)	0.0151 (8)	0.0232 (8)
O7	0.0391 (10)	0.0592 (12)	0.0405 (10)	0.0248 (9)	0.0139 (8)	0.0150 (8)
O8	0.0446 (11)	0.0642 (12)	0.0342 (10)	0.0213 (10)	0.0160 (8)	0.0187 (8)

### Geometric parameters (Å, °)

**Table:**

C1—O1	1.430 (3)	C32—O6	1.432 (3)
C1—C2	1.515 (4)	C32—C33	1.516 (4)
C1—H1A	0.9700	C32—H32A	0.9700
C1—H1B	0.9700	C32—H32B	0.9700
C2—C3	1.519 (3)	C33—C36	1.514 (4)
C2—C4	1.521 (4)	C33—C34	1.526 (4)
C2—C5	1.526 (3)	C33—C35	1.528 (3)
C3—O2	1.429 (3)	C34—O5	1.428 (3)
C3—H3A	0.9700	C34—H34A	0.9700
C3—H3B	0.9700	C34—H34B	0.9700
C4—O4	1.438 (3)	C35—O7	1.434 (3)
C4—H4A	0.9700	C35—H35A	0.9700
C4—H4B	0.9700	C35—H35B	0.9700
C5—O3	1.423 (3)	C36—O8	1.432 (3)
C5—H5A	0.9700	C36—H36A	0.9700
C5—H5B	0.9700	C36—H36B	0.9700
C6—O2	1.420 (3)	C37—O5	1.425 (3)
C6—O1	1.421 (3)	C37—O6	1.427 (3)
C6—C7	1.517 (4)	C37—C38	1.523 (4)
C6—C13	1.530 (3)	C37—C44	1.530 (3)
C7—C12	1.380 (4)	C38—C43	1.386 (4)
C7—C8	1.387 (4)	C38—C39	1.388 (4)
C8—C9	1.390 (4)	C39—C40	1.373 (4)
C8—H8	0.9300	C39—H39	0.9300
C9—C10	1.345 (5)	C40—C41	1.375 (5)
C9—H9	0.9300	C40—H40	0.9300
C10—C11	1.374 (5)	C41—C42	1.370 (4)
C10—H10	0.9300	C41—H41	0.9300
C11—C12	1.386 (5)	C42—C43	1.381 (4)
C11—H11	0.9300	C42—H42	0.9300
C12—H12	0.9300	C43—H43	0.9300
C13—C18	1.385 (4)	C44—C45	1.380 (4)
C13—C14	1.385 (4)	C44—C49	1.383 (4)
C14—C15	1.386 (4)	C45—C46	1.385 (4)
C14—H14	0.9300	C45—H45	0.9300
C15—C16	1.364 (5)	C46—C47	1.378 (5)
C15—H15	0.9300	C46—H46	0.9300
C16—C17	1.377 (5)	C47—C48	1.370 (5)
C16—H16	0.9300	C47—H47	0.9300
C17—C18	1.384 (4)	C48—C49	1.383 (4)
C17—H17	0.9300	C48—H48	0.9300
C18—H18	0.9300	C49—H49	0.9300
C19—O3	1.410 (3)	C50—O7	1.415 (3)
C19—O4	1.433 (3)	C50—O8	1.433 (3)
C19—C20	1.524 (4)	C50—C57	1.517 (4)
C19—C26	1.531 (4)	C50—C51	1.534 (4)
C20—C21	1.383 (4)	C51—C56	1.381 (4)
C20—C25	1.383 (4)	C51—C52	1.385 (4)
C21—C22	1.387 (4)	C52—C53	1.370 (4)
C21—H21	0.9300	C52—H52	0.9300
C22—C23	1.373 (4)	C53—C54	1.371 (4)
C22—H22	0.9300	C53—H53	0.9300
C23—C24	1.369 (4)	C54—C55	1.360 (4)
C23—H23	0.9300	C54—H54	0.9300
C24—C25	1.389 (4)	C55—C56	1.385 (4)
C24—H24	0.9300	C55—H55	0.9300
C25—H25	0.9300	C56—H56	0.9300
C26—C27	1.375 (4)	C57—C58	1.391 (4)
C26—C31	1.394 (4)	C57—C62	1.393 (4)
C27—C28	1.395 (4)	C58—C59	1.392 (4)
C27—H27	0.9300	C58—H58	0.9300
C28—C29	1.372 (5)	C59—C60	1.355 (5)
C28—H28	0.9300	C59—H59	0.9300
C29—C30	1.361 (5)	C60—C61	1.378 (4)
C29—H29	0.9300	C60—H60	0.9300
C30—C31	1.385 (4)	C61—C62	1.381 (4)
C30—H30	0.9300	C61—H61	0.9300
C31—H31	0.9300	C62—H62	0.9300
			
O1—C1—C2	110.7 (2)	C33—C32—H32B	109.4
O1—C1—H1A	109.5	H32A—C32—H32B	108.0
C2—C1—H1A	109.5	C36—C33—C32	110.7 (2)
O1—C1—H1B	109.5	C36—C33—C34	110.7 (2)
C2—C1—H1B	109.5	C32—C33—C34	106.9 (2)
H1A—C1—H1B	108.1	C36—C33—C35	106.4 (2)
C1—C2—C3	106.6 (2)	C32—C33—C35	111.1 (2)
C1—C2—C4	111.1 (2)	C34—C33—C35	111.0 (2)
C3—C2—C4	110.9 (2)	O5—C34—C33	111.1 (2)
C1—C2—C5	111.7 (2)	O5—C34—H34A	109.4
C3—C2—C5	110.9 (2)	C33—C34—H34A	109.4
C4—C2—C5	105.8 (2)	O5—C34—H34B	109.4
O2—C3—C2	110.0 (2)	C33—C34—H34B	109.4
O2—C3—H3A	109.7	H34A—C34—H34B	108.0
C2—C3—H3A	109.7	O7—C35—C33	110.8 (2)
O2—C3—H3B	109.7	O7—C35—H35A	109.5
C2—C3—H3B	109.7	C33—C35—H35A	109.5
H3A—C3—H3B	108.2	O7—C35—H35B	109.5
O4—C4—C2	110.7 (2)	C33—C35—H35B	109.5
O4—C4—H4A	109.5	H35A—C35—H35B	108.1
C2—C4—H4A	109.5	O8—C36—C33	111.2 (2)
O4—C4—H4B	109.5	O8—C36—H36A	109.4
C2—C4—H4B	109.5	C33—C36—H36A	109.4
H4A—C4—H4B	108.1	O8—C36—H36B	109.4
O3—C5—C2	110.47 (19)	C33—C36—H36B	109.4
O3—C5—H5A	109.6	H36A—C36—H36B	108.0
C2—C5—H5A	109.6	O5—C37—O6	110.10 (19)
O3—C5—H5B	109.6	O5—C37—C38	105.9 (2)
C2—C5—H5B	109.6	O6—C37—C38	105.07 (19)
H5A—C5—H5B	108.1	O5—C37—C44	112.3 (2)
O2—C6—O1	111.09 (19)	O6—C37—C44	111.0 (2)
O2—C6—C7	106.6 (2)	C38—C37—C44	112.1 (2)
O1—C6—C7	105.5 (2)	C43—C38—C39	117.3 (3)
O2—C6—C13	111.4 (2)	C43—C38—C37	122.5 (2)
O1—C6—C13	111.83 (19)	C39—C38—C37	120.2 (2)
C7—C6—C13	110.1 (2)	C40—C39—C38	121.2 (3)
C12—C7—C8	118.3 (3)	C40—C39—H39	119.4
C12—C7—C6	121.1 (3)	C38—C39—H39	119.4
C8—C7—C6	120.6 (2)	C39—C40—C41	120.7 (3)
C7—C8—C9	120.5 (3)	C39—C40—H40	119.7
C7—C8—H8	119.7	C41—C40—H40	119.7
C9—C8—H8	119.7	C42—C41—C40	119.1 (3)
C10—C9—C8	119.9 (3)	C42—C41—H41	120.4
C10—C9—H9	120.0	C40—C41—H41	120.4
C8—C9—H9	120.0	C41—C42—C43	120.2 (3)
C9—C10—C11	121.1 (3)	C41—C42—H42	119.9
C9—C10—H10	119.4	C43—C42—H42	119.9
C11—C10—H10	119.4	C42—C43—C38	121.4 (3)
C10—C11—C12	119.3 (3)	C42—C43—H43	119.3
C10—C11—H11	120.4	C38—C43—H43	119.3
C12—C11—H11	120.4	C45—C44—C49	119.1 (2)
C7—C12—C11	120.9 (3)	C45—C44—C37	121.2 (2)
C7—C12—H12	119.6	C49—C44—C37	119.3 (2)
C11—C12—H12	119.6	C44—C45—C46	120.3 (3)
C18—C13—C14	118.8 (3)	C44—C45—H45	119.8
C18—C13—C6	120.0 (2)	C46—C45—H45	119.8
C14—C13—C6	120.9 (3)	C47—C46—C45	120.0 (3)
C13—C14—C15	120.4 (3)	C47—C46—H46	120.0
C13—C14—H14	119.8	C45—C46—H46	120.0
C15—C14—H14	119.8	C48—C47—C46	119.9 (3)
C16—C15—C14	120.1 (3)	C48—C47—H47	120.0
C16—C15—H15	119.9	C46—C47—H47	120.0
C14—C15—H15	119.9	C47—C48—C49	120.2 (3)
C15—C16—C17	120.3 (3)	C47—C48—H48	119.9
C15—C16—H16	119.9	C49—C48—H48	119.9
C17—C16—H16	119.9	C48—C49—C44	120.4 (3)
C16—C17—C18	119.9 (3)	C48—C49—H49	119.8
C16—C17—H17	120.0	C44—C49—H49	119.8
C18—C17—H17	120.0	O7—C50—O8	110.7 (2)
C17—C18—C13	120.4 (3)	O7—C50—C57	111.2 (2)
C17—C18—H18	119.8	O8—C50—C57	111.4 (2)
C13—C18—H18	119.8	O7—C50—C51	106.1 (2)
O3—C19—O4	111.13 (19)	O8—C50—C51	104.26 (19)
O3—C19—C20	107.2 (2)	C57—C50—C51	112.8 (2)
O4—C19—C20	105.10 (19)	C56—C51—C52	118.0 (3)
O3—C19—C26	110.9 (2)	C56—C51—C50	121.9 (2)
O4—C19—C26	111.2 (2)	C52—C51—C50	120.1 (2)
C20—C19—C26	111.2 (2)	C53—C52—C51	120.9 (3)
C21—C20—C25	119.1 (3)	C53—C52—H52	119.5
C21—C20—C19	120.4 (2)	C51—C52—H52	119.5
C25—C20—C19	120.6 (3)	C52—C53—C54	120.3 (3)
C20—C21—C22	120.1 (3)	C52—C53—H53	119.8
C20—C21—H21	119.9	C54—C53—H53	119.8
C22—C21—H21	119.9	C55—C54—C53	119.9 (3)
C23—C22—C21	120.6 (3)	C55—C54—H54	120.1
C23—C22—H22	119.7	C53—C54—H54	120.1
C21—C22—H22	119.7	C54—C55—C56	120.1 (3)
C24—C23—C22	119.5 (3)	C54—C55—H55	120.0
C24—C23—H23	120.3	C56—C55—H55	120.0
C22—C23—H23	120.3	C51—C56—C55	120.8 (3)
C23—C24—C25	120.6 (3)	C51—C56—H56	119.6
C23—C24—H24	119.7	C55—C56—H56	119.6
C25—C24—H24	119.7	C58—C57—C62	118.2 (3)
C20—C25—C24	120.1 (3)	C58—C57—C50	120.4 (2)
C20—C25—H25	119.9	C62—C57—C50	121.2 (2)
C24—C25—H25	119.9	C57—C58—C59	120.0 (3)
C27—C26—C31	118.3 (3)	C57—C58—H58	120.0
C27—C26—C19	122.6 (2)	C59—C58—H58	120.0
C31—C26—C19	119.0 (3)	C60—C59—C58	120.8 (3)
C26—C27—C28	121.1 (3)	C60—C59—H59	119.6
C26—C27—H27	119.5	C58—C59—H59	119.6
C28—C27—H27	119.5	C59—C60—C61	120.3 (3)
C29—C28—C27	119.6 (4)	C59—C60—H60	119.9
C29—C28—H28	120.2	C61—C60—H60	119.9
C27—C28—H28	120.2	C60—C61—C62	119.7 (3)
C30—C29—C28	120.1 (3)	C60—C61—H61	120.1
C30—C29—H29	120.0	C62—C61—H61	120.1
C28—C29—H29	120.0	C61—C62—C57	121.0 (3)
C29—C30—C31	120.7 (3)	C61—C62—H62	119.5
C29—C30—H30	119.7	C57—C62—H62	119.5
C31—C30—H30	119.7	C6—O1—C1	114.66 (19)
C30—C31—C26	120.2 (3)	C6—O2—C3	113.56 (18)
C30—C31—H31	119.9	C19—O3—C5	113.95 (19)
C26—C31—H31	119.9	C19—O4—C4	114.20 (19)
O6—C32—C33	111.3 (2)	C37—O5—C34	114.3 (2)
O6—C32—H32A	109.4	C37—O6—C32	113.83 (18)
C33—C32—H32A	109.4	C50—O7—C35	114.6 (2)
O6—C32—H32B	109.4	C36—O8—C50	113.54 (19)
			
O1—C1—C2—C3	−55.4 (3)	O6—C37—C38—C39	−41.2 (3)
O1—C1—C2—C4	−176.4 (2)	C44—C37—C38—C39	−161.9 (2)
O1—C1—C2—C5	65.8 (3)	C43—C38—C39—C40	−0.8 (4)
C1—C2—C3—O2	57.3 (3)	C37—C38—C39—C40	178.7 (3)
C4—C2—C3—O2	178.34 (19)	C38—C39—C40—C41	0.0 (5)
C5—C2—C3—O2	−64.4 (3)	C39—C40—C41—C42	1.7 (5)
C1—C2—C4—O4	−65.5 (3)	C40—C41—C42—C43	−2.5 (5)
C3—C2—C4—O4	176.18 (19)	C41—C42—C43—C38	1.7 (5)
C5—C2—C4—O4	55.9 (3)	C39—C38—C43—C42	0.0 (4)
C1—C2—C5—O3	63.5 (3)	C37—C38—C43—C42	−179.5 (3)
C3—C2—C5—O3	−177.9 (2)	O5—C37—C44—C45	23.1 (3)
C4—C2—C5—O3	−57.5 (3)	O6—C37—C44—C45	146.9 (2)
O2—C6—C7—C12	32.5 (3)	C38—C37—C44—C45	−95.9 (3)
O1—C6—C7—C12	150.7 (2)	O5—C37—C44—C49	−163.8 (2)
C13—C6—C7—C12	−88.5 (3)	O6—C37—C44—C49	−40.0 (3)
O2—C6—C7—C8	−147.6 (2)	C38—C37—C44—C49	77.2 (3)
O1—C6—C7—C8	−29.4 (3)	C49—C44—C45—C46	1.6 (4)
C13—C6—C7—C8	91.4 (3)	C37—C44—C45—C46	174.7 (3)
C12—C7—C8—C9	0.2 (4)	C44—C45—C46—C47	−1.7 (5)
C6—C7—C8—C9	−179.7 (2)	C45—C46—C47—C48	0.7 (5)
C7—C8—C9—C10	−1.0 (4)	C46—C47—C48—C49	0.3 (5)
C8—C9—C10—C11	1.3 (5)	C47—C48—C49—C44	−0.4 (4)
C9—C10—C11—C12	−0.8 (6)	C45—C44—C49—C48	−0.5 (4)
C8—C7—C12—C11	0.4 (5)	C37—C44—C49—C48	−173.8 (2)
C6—C7—C12—C11	−179.7 (3)	O7—C50—C51—C56	−147.5 (2)
C10—C11—C12—C7	−0.1 (5)	O8—C50—C51—C56	95.5 (3)
O2—C6—C13—C18	−34.1 (3)	C57—C50—C51—C56	−25.5 (3)
O1—C6—C13—C18	−159.0 (2)	O7—C50—C51—C52	35.5 (3)
C7—C6—C13—C18	84.0 (3)	O8—C50—C51—C52	−81.5 (3)
O2—C6—C13—C14	153.1 (2)	C57—C50—C51—C52	157.6 (2)
O1—C6—C13—C14	28.2 (3)	C56—C51—C52—C53	−0.1 (4)
C7—C6—C13—C14	−88.8 (3)	C50—C51—C52—C53	177.0 (2)
C18—C13—C14—C15	0.7 (4)	C51—C52—C53—C54	0.5 (5)
C6—C13—C14—C15	173.7 (3)	C52—C53—C54—C55	−1.2 (5)
C13—C14—C15—C16	−0.4 (5)	C53—C54—C55—C56	1.4 (5)
C14—C15—C16—C17	−0.5 (5)	C52—C51—C56—C55	0.4 (4)
C15—C16—C17—C18	1.1 (5)	C50—C51—C56—C55	−176.6 (3)
C16—C17—C18—C13	−0.7 (5)	C54—C55—C56—C51	−1.1 (5)
C14—C13—C18—C17	−0.2 (4)	O7—C50—C57—C58	32.1 (3)
C6—C13—C18—C17	−173.2 (3)	O8—C50—C57—C58	156.1 (2)
O3—C19—C20—C21	16.2 (3)	C51—C50—C57—C58	−87.0 (3)
O4—C19—C20—C21	−102.1 (3)	O7—C50—C57—C62	−152.1 (2)
C26—C19—C20—C21	137.6 (2)	O8—C50—C57—C62	−28.1 (3)
O3—C19—C20—C25	−165.6 (2)	C51—C50—C57—C62	88.7 (3)
O4—C19—C20—C25	76.1 (3)	C62—C57—C58—C59	0.4 (4)
C26—C19—C20—C25	−44.2 (3)	C50—C57—C58—C59	176.2 (3)
C25—C20—C21—C22	0.3 (4)	C57—C58—C59—C60	0.4 (5)
C19—C20—C21—C22	178.6 (2)	C58—C59—C60—C61	−1.8 (5)
C20—C21—C22—C23	−0.3 (4)	C59—C60—C61—C62	2.5 (5)
C21—C22—C23—C24	−0.1 (5)	C60—C61—C62—C57	−1.8 (4)
C22—C23—C24—C25	0.4 (5)	C58—C57—C62—C61	0.3 (4)
C21—C20—C25—C24	0.0 (4)	C50—C57—C62—C61	−175.5 (3)
C19—C20—C25—C24	−178.3 (3)	O2—C6—O1—C1	−52.8 (3)
C23—C24—C25—C20	−0.3 (5)	C7—C6—O1—C1	−168.03 (19)
O3—C19—C26—C27	−144.8 (2)	C13—C6—O1—C1	72.3 (3)
O4—C19—C26—C27	−20.6 (3)	C2—C1—O1—C6	55.2 (3)
C20—C19—C26—C27	96.1 (3)	O1—C6—O2—C3	54.6 (3)
O3—C19—C26—C31	39.5 (3)	C7—C6—O2—C3	169.05 (19)
O4—C19—C26—C31	163.6 (2)	C13—C6—O2—C3	−70.8 (2)
C20—C19—C26—C31	−79.6 (3)	C2—C3—O2—C6	−58.7 (2)
C31—C26—C27—C28	0.2 (4)	O4—C19—O3—C5	−54.4 (3)
C19—C26—C27—C28	−175.5 (3)	C20—C19—O3—C5	−168.66 (19)
C26—C27—C28—C29	−0.6 (5)	C26—C19—O3—C5	69.8 (2)
C27—C28—C29—C30	1.0 (5)	C2—C5—O3—C19	59.0 (3)
C28—C29—C30—C31	−1.1 (5)	O3—C19—O4—C4	52.7 (3)
C29—C30—C31—C26	0.8 (5)	C20—C19—O4—C4	168.2 (2)
C27—C26—C31—C30	−0.3 (4)	C26—C19—O4—C4	−71.4 (3)
C19—C26—C31—C30	175.6 (3)	C2—C4—O4—C19	−55.7 (3)
O6—C32—C33—C36	174.8 (2)	O6—C37—O5—C34	−55.3 (3)
O6—C32—C33—C34	54.1 (3)	C38—C37—O5—C34	−168.41 (19)
O6—C32—C33—C35	−67.2 (3)	C44—C37—O5—C34	68.9 (3)
C36—C33—C34—O5	−174.3 (2)	C33—C34—O5—C37	56.6 (3)
C32—C33—C34—O5	−53.6 (3)	O5—C37—O6—C32	55.4 (3)
C35—C33—C34—O5	67.7 (3)	C38—C37—O6—C32	168.99 (19)
C36—C33—C35—O7	−54.7 (3)	C44—C37—O6—C32	−69.6 (2)
C32—C33—C35—O7	−175.2 (2)	C33—C32—O6—C37	−57.2 (3)
C34—C33—C35—O7	65.9 (3)	O8—C50—O7—C35	−54.0 (3)
C32—C33—C36—O8	176.61 (19)	C57—C50—O7—C35	70.3 (3)
C34—C33—C36—O8	−65.0 (3)	C51—C50—O7—C35	−166.62 (19)
C35—C33—C36—O8	55.7 (3)	C33—C35—O7—C50	56.3 (3)
O5—C37—C38—C43	−105.2 (3)	C33—C36—O8—C50	−57.6 (3)
O6—C37—C38—C43	138.3 (2)	O7—C50—O8—C36	54.4 (3)
C44—C37—C38—C43	17.6 (3)	C57—C50—O8—C36	−69.9 (3)
O5—C37—C38—C39	75.3 (3)	C51—C50—O8—C36	168.1 (2)

### Hydrogen-bond geometry (Å, °)

**Table:**

*D*—H···*A*	*D*—H	H···*A*	*D*···*A*	*D*—H···*A*
C9—H9···Cg1^i^	0.93	2.83	3.722 (2)	161
C30—H30···Cg2^ii^	0.93	2.84	3.523 (3)	131
C3—H3A···O6^ii^	0.97	2.53	3.436 (3)	156

Symmetry codes: (i) −*x*+1, −*y*, −*z*+1; (ii) −*x*+1, −*y*+1, −*z*+1.
